# Alcohol and Hypertension—A Fresh Look on the Mechanistic Aspects of a Fatal Epidemiologic Relationship

**DOI:** 10.3390/biom16091269

**Published:** 2026-09-02

**Authors:** Klaus Groschner, Ichiro Wakabayashi

**Affiliations:** 1 Division of Medical Physics and Biophysics, Gottfried Schatz Research Center, Medical University of Graz, 8010 Graz, Austria; 2 Department of Preventive Medicine, School of Medicine, Hyogo Medical University, Nishinomiya 663-8501, Hyogo, Japan; wakabaya@hyo-med.ac.jp

**Keywords:** alcohol associated diseases, arterial hypertension, ethanol, ion channels, protein–lipid interactions

## Abstract

Arterial hypertension is a key trigger for all major, life-threatening cardiovascular events such as arrhythmia, stroke, cardiac infarction and heart failure. Ethanol consumption has been identified as a critical risk factor, which promotes hypertension and thereby corrupts cardiovascular health and limits overall life expectancy. For heavy drinkers (≥72 g/day), reduction in half of the alcohol consumption reportedly results in considerable decreases in systolic and diastolic blood pressure levels amounting to 5.50 and 3.97 mmHg, respectively. Based on the complex network of ethanol effects on mammalian physiology, diverse mechanistic concepts have been postulated for a causal linkage between alcohol consumption and distorted blood pressure regulation. The impact of ethanol on the cardiovascular system involves a wide range of ethanol-sensitive cell types, ranging from neurons to vascular smooth muscle, endothelial and immune-cells, all of which express an interdependent array of potential ethanol target structures. Here, we provide an update on currently available evidence for a causal link between alcohol consumption and the etiology of arterial hypertension. The potential role of ethanol target structures with particular focus on membrane ion channels is discussed along with novel concepts of lipid-dependent modulation of signaling processes in the plasma membrane by ethanol.

## 1. Epidemiology of Alcohol-Induced Hypertension

Habitual alcohol consumption diversely affects cardiovascular health depending on amount of alcohol. The risks of coronary artery disease and ischemic stroke are lower in light-to-moderate drinkers than in nondrinkers [1], which is mainly explained by alcohol-induced elevation of blood HDL cholesterol and suppression of blood coagulation [2]. On the contrary, the risk of cardiovascular disease, especially that of hemorrhagic stroke, is higher in heavy drinkers than in nondrinkers [3], in which alcohol-induced hypertension is greatly involved [4].

There has been an accumulation of knowledge on the relationship between alcohol and blood pressure. In the previous studies of meta-analysis (Table 1), a highly consistent, dose-dependent positive association has been shown between alcohol consumption and incident hypertension in men [5,6,7,8,9,10]. The high consistency of dose-dependent positive association (Table 1) is striking in view of the known various intrinsic and extrinsic factors, such as chronic mental stress, neurohumoral activation, oxidative stress, inflammation and autonomic dysfunction, that contribute to the development of hypertension. Of note, there is a non-linear J-shaped association between alcohol consumption and cardiovascular disease, especially coronary heart disease. Hypertension and dyslipidemia are major cardiovascular risk factors. Alcohol consumption is associated with lower LDL cholesterol level and higher HDL cholesterol level. Thus, it has been suggested that alcohol exerts both beneficial and detrimental effects on cardiovascular health, which may explain the J-shaped association between alcohol consumption and cardiovascular disease.

In women, compared with nondrinkers, the incidence of hypertension has been shown to be lower [5,6] or not significantly different [7,8,9] in light drinkers (5~10 g ethanol per day), but to be higher in heavy drinkers [9]. Thus, there is a gender-related difference in alcohol–blood pressure relationship such as a linear positive relationship in men vs. a J-shaped relationship in women [10]. The reason for this difference is unknown but might be explained by anti-hypertensive effect of estrogen [11]. An ethnic difference in the sensitivity of elevation of blood pressure to alcohol has also been suggested in the previous studies of meta-analysis, although their findings were not consistent: the sensitivity was higher in Asian men than in western men [5,8] and was higher in black people compared with white people and Asians [10] or was higher in white people than in black people [9]. There has also been an accumulation of knowledge on the effect of reduction in alcohol consumption on blood pressure. According to the meta-analysis using 36 clinical trials [12], there was a significant association between alcohol reduction and decreased blood pressure in drinkers with an average daily alcohol consumption of more than 24 g: mean average decreases in systolic and diastolic blood pressure were 5.50 and 3.97 mmHg, respectively, when alcohol consumption was reduced by half in drinkers with an average daily alcohol consumption of 72 g or higher. Thus, alcohol consumption is confirmed as a risk factor for hypertension, which is deeply involved in the pathogenesis of cardiovascular disease.

Although the association between alcohol and hypertension was first reported more than a century ago, this finding had been almost ignored for a long time, as reviewed in [13]. Systematic, population-based, epidemiological research on this topic started in the 1970s including a groundbreaking Kaiser Permanente cohort study by Klatsky et al. (1977; [14]). Nowadays, habitual alcohol consumption is confirmed as a risk factor of chronic hypertension, as mentioned above. However, the exact mechanisms underlying this relationship still appear enigmatic and remain to be clarified.

## 2. Regulatory Pathways and Cell Types Involved in Alcohol-Induced Hypertension

Like many other physiological parameters which are affected by alcohol consumption, BP is modulated in a complex and multimodal manner. As outlined above, the dose of ethanol consumption is a complex determinant of the cardiovascular outcome both in an acute and a chronic pathological setting [15]. A variety of cellular and molecular targets have been suggested to explain acute as well as chronic effects of ethanol intake on blood pressure and divergent concepts have been put forward to explain drinking-associated promotion of hypertension [16,17,18,19]. Of note, this applies similarly for all other alcohol-related cardiovascular disorders. All etiologies of alcohol-associated diseases appear multifactorial in nature. Modulatory effects of ethanol on the cardiovascular system involves an array of highly ethanol-sensing cell types, ranging from neurons to vascular smooth muscle, endothelial and immune cells. Of note, also cells which are less extensively characterized but emerging key players in cardiovascular function and pathology such as perivascular adipocytes [20,21,22,23]. For most of these cell types the expression of predicted or already characterized ethanol target molecules (“ethanol receptors”) has been reported. Most of the to-date identified ethanol receptors such as K^+^ channels and TRP channels (discussed in detail below) are widely expressed in the central nervous system as well as the vascular bed. Therefore, these target structures may play a complex and multifunctional role in alcohol-induced hypertension.

**Figure 1:**
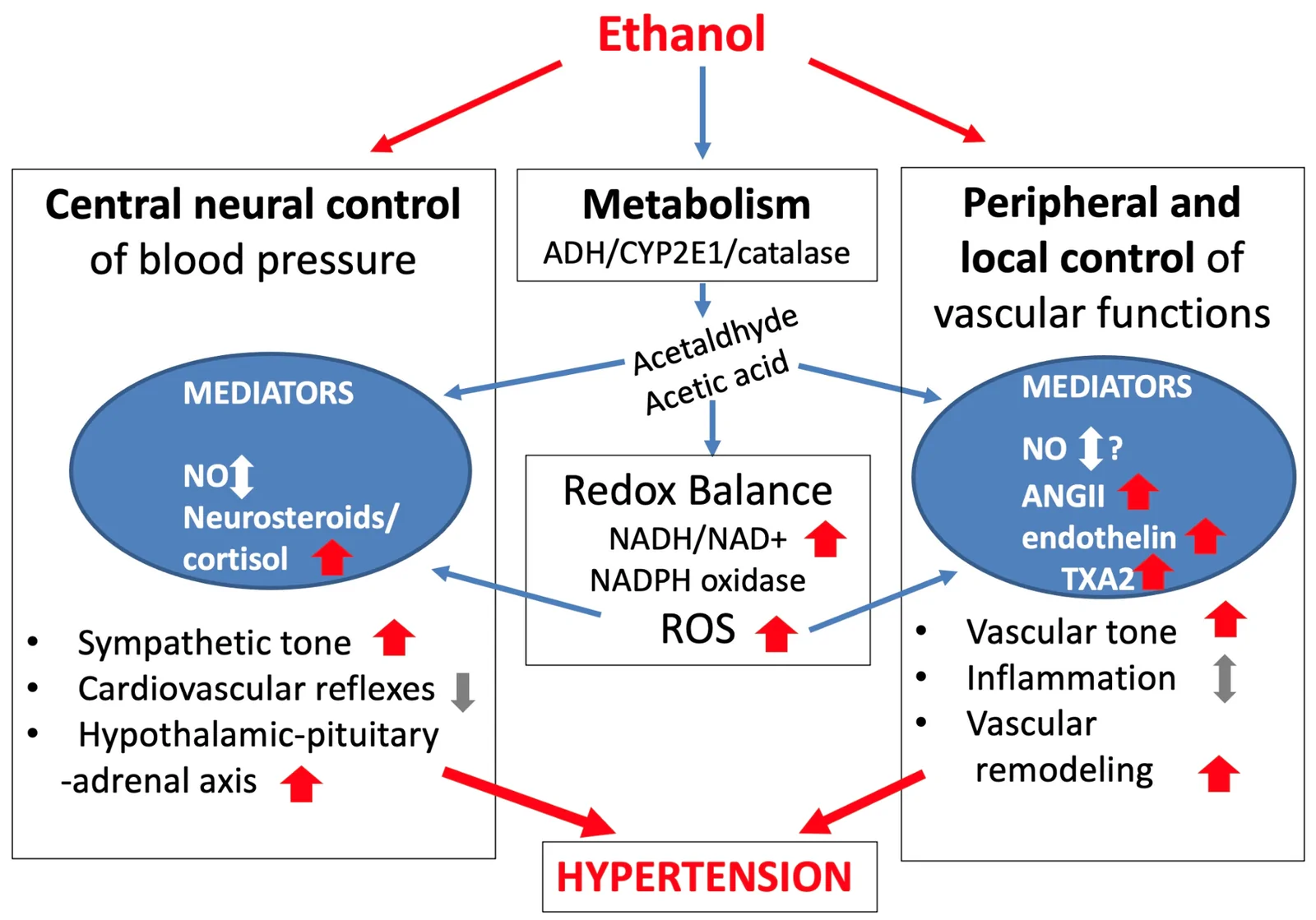
Multiple pathways of organic dysfunction leading to alcohol-associated hypertension. A large body of evidence has been accumulated for the targeting of central and peripheral blood pressure control mechanisms by acute or chronic alcohol consumption This scheme illustrates the currently considered divergent concepts of drinking-associated hypertension development with respect to the hypothesized primary site(s) of ethanol action. Plausible mechanisms to date suggested as the basis of ethanol-induced hypertension include its interference with the central blood pressure control (**left**) and/or the disruption of peripheral blood vessel function (**right**). These mechanisms are likely to overlap as most of the proposed molecular targets and ethanol-sensitive signaling pathways are expressed in both the central nervous systems and the peripheral vascular bed. This principle holds true also for the aspect of ethanol actions that are generated by products of ethanol metabolism (**middle**). The scheme illustrates the overlap and potential synergy of a selection of the currently proposed pathomechanisms but does not represent a comprehensive overview of mechanistic aspects.

For the effects of ethanol on blood pressure, one might group the underlying pathomechanisms in (i) effects on the central neural control of blood pressure including the autonomous nervous system, with altered cardiovascular reflexes and increased sympathetic activity [24,25] and as-yet-unclear involvement of the hypothalamic–pituitary–adrenal (HPA) axis as a primary target structure of ethanol action [26,27], and (ii) impairment of local blood pressure control at the level of peripheral mediator generation and/or direct interference with vascular cell functions (Figure 1) [25,28,29,30]. Of note, these mechanisms are tightly interrelated and cannot be unambiguously separated. Ethanol is metabolized by a range of enzymes, most prominently by ADH, ALDH, catalase and CYP2E1 [31] but also by phospholipase D (PLD) [32,33]. Importantly, ethanol metabolites, specifically acetaldehyde but also reactive oxygen species as a byproduct of ethanol metabolism, are considered to significantly interfere with vascular homeostasis [34,35,36], presumably due to de-arrangement of vascular metabolic signaling and perturbance of local mediators production resulting in vascular hyperreactivity. In the brain, acetaldehyde exerts neurotoxicity presumably via generation of reactive oxygen species based on an impairment of mitochondrial function [37]. Similarly, ethanol metabolism by CYP2E1 is considered of pathological relevance due to generation of reactive oxygen species [38]. The pivotal role of ethanol-induced oxidative stress has been extensively documented and is considered to involve divergent and potentially synergistic pathways including mitochondrial dysregulation [39], enhanced NADPH oxidase (NOX) activity [36] and CYP2E1 metabolism [38].

More recently, the formation of lipid–alcohol metabolites such as phosphatidylethanol, generated by PLD, has been implicated in the functional consequences of alcohol intoxication [33,40]. Of note, phosphatidylethanol represents a well-established biomarker of alcohol use disorders [41,42]. Interestingly, PLD-generated transphosphorylation products have recently been shown to interfere with the function of membrane proteins, disrupting regulatory protein–lipid interactions (see in more detail below).

The causal chain of events leading to hypertension in response to chronic alcohol consumption is likely to involve a multiple-signaling mechanism that is modified sequentially with a certain temporal choreography. A typical example for such a potential “hypertensive chain of events” is the initiation of enhanced sympathetic nerve activity [43,44] triggered in part by an initial drop in blood pressure as an acute effect of alcohol consumption, based on direct effects of ethanol on vascular contractility. This trigger event is likely paralleled by the direct impact of ethanol on central and peripheral neurons leading to increased sympathetic tone [43,44] and the activation of the renin–angiotensin system (RAS) [28,45,46] as well as the downregulation of baroreceptor reflexes [47,48]. As a consequence of RAS activation, enhanced angiotensin II (ANGII) levels represent one typical event that inevitably initiates maladaptive tissue remodeling, promotes oxidative stress and accelerates the development of hypertension. Some of the processes, which have been implicated in alcohol drinking-associated hypertension such as ethanol-induced activation of the HPA axis with elevation of cortisol and neurosteroids, tightly overlap with psychopharmacological aspects and drinking behavior [49].

There is a wide consensus with respect to the concept of alcohol-induced hypertension being triggered primarily and for a large part by sympathetic hyperactivation associated with enhanced RAS activity, oxidative stress responses and concurrent activation of the HPA axis. Alcohol consumption has been convincingly correlated with enhanced circulating levels of catecholamines, renin, angiotensin I and II as well as aldosterone [50] (reviewed in [36]. Despite a vast body of evidence for the actions of ethanol and/or its metabolites on distinct regulatory pathways involved in cardiovascular homeostasis and blood pressure control, there is still little knowledge on the causal relations between these events. Pharmacological evidence suggests that sympathetic dysregulation may be upstream of RAS activation [51], which in turn is an upstream signal for enhanced production of reactive oxygen species leading to vascular dysfunction and remodeling. Nonetheless, the underlying molecular mechanisms, primary target structures and potential causal relations and synergies between individual pathways remain ill defined.

Hence, the main challenge to advance the field is to gain a deeper understanding of the causal chains of molecular and cellular events involved in ethanol-triggered diseases. Moreover, a quantitative description of these pathways is needed to generate useful computational models of the multifactorial, pathogenetic network underlying ethanol-triggered pathologies. This may be achieved by future AI-supported approaches. Importantly, a prerequisite for this goal, as well as a promising additional strategy to establish targeted therapies and preventive strategies, is the analysis of the biological actions of ethanol and its metabolites at the molecular level. Identification of pharmacological concepts, which are common for a set or even a wider range of primary ethanol targets, is expected to pave the way towards novel clinical interventions.

The aim of this perspectives review is to provide an overview on current state of the art with focus on the emerging concept of ethanol-induced disruption of regulatory lipid–protein switches as a basis of ethanol toxicity and alcohol-induced hypertension.

## 3. Molecular Mechanism of Alcohol-Induced Hypertension—A Focus on Membrane-Delimited Signaling Processes

There is a long-standing consensus about the importance of ethanol partitioning into cell membranes [52] as a basis of nonspecific (lipid bilayer centric) [53] but also specific (protein centric) actions on membrane proteins. It appears plausible to consider transmembrane signaling molecules and membrane-delimited signaling pathways as key targets of ethanol action.

Current mechanistic concepts at the molecular level assume that ethanol intake leads to interference with signal transduction processes by direct interaction of ethanol with specific alcohol-recognition domains (hydrophobic alcohol-binding pockets) in signaling proteins and/or by a rather specific disruption of regulatory protein–lipid interactions as convincingly exemplified in different K^+^ channels [54,55]. Besides its potential role as a direct pharmaco-/toxicological ligand for signaling molecules (“alcohol receptors”), ethanol is considered to exert significant impact on cell functions as a consequence of its metabolism involving the action of ethanol metabolites [17,56]. Nonetheless, over the past decades, an impressive array of ethanol-sensitive signaling molecules has been identified and the interaction of alcohols with these target structures has extensively been characterized. This search for “alcohol receptors” identified various ion channels as biomolecules, which harbor distinct alcohol (ethanol)-binding sites [57,58,59,60]. Again, it is important to note that ethanol and other alcohols have long been recognized to modulate cellular functions by less specific interference with physicochemical and structural features of cell membranes [53], thereby imposing profound effects on membrane-resident signaling molecules. In addition, metabolites of ethanol such as acetaldehyde [61], acetate [62] and phosphatitylethanol [63] have been identified as modulators of membrane-delimited signaling processes. Hence, the impact of ethanol on cell and tissue functions is commonly understood to originate from either (i) direct, reversible binding of ethanol to specific molecular targets, (ii) the action of active ethanol metabolites on these targets, or (iii) partitioning of ethanol into biomembranes leading to disturbances in lipid bilayer architecture and microdomain organization of target signaling molecules. By way of these different mechanisms, ethanol affects a large array of membrane-resident signaling systems and regulatory pathways, for which a linkage to the etiology of arterial hypertension is plausible and/or documented [64,65,66]. Delineation of the relative importance and potential synergy of the identified ethanol-sensitive signaling pathways with respect to hypertension development is to date barely feasible. Nonetheless, it appears rewarding to further analyze the molecular mechanisms by which ethanol interferes with blood pressure regulation and vascular homeostasis, since this may unravel new therapeutic targets and support advances in the development of cardiovascular therapies. Despite a lack of comprehensive mechanistic concepts for the etiology of ethanol-induced pathologies, there is consensus about a key role of membrane-resident signaling processes. In the brain, ethanol was found to affect the neurotransmitter release machinery [67] and to severely interfere with membrane transport processes which govern electrical excitability and/or electrogenic signaling processes [68]. Based on current knowledge, ethanol and/or its metabolites act as pathogenic modulators of ion transport across cell membranes and affect critical ion transport systems either via direct reversible binding, interference with expression of transport molecules or disruption of their membrane bilayer organization. In view of the well-established role of neuronal circuits in the pathogenesis of hypertension, ion channels appear as key targets of ethanol action. Specifically, the brain ion channels are well recognized as a group of primary alcohol target sites [68]. The direct binding of ethanol to pentameric ligand-gated channels represents a paradigm mechanism underlying ethanol action in the central nervous system [57]. Peripheral vascular functionality and pathological remodeling are also critically dependent on plasma membrane ion transport involving ion channels and associated signaling molecules that are ethanol sensitive [23,36,69]. We therefore decided to provide here an overview of emerging concepts describing ethanol modulation of ion channel function and their relevance for hypertension development. Below we will focus on ion channels, which are involved in blood pressure control and considered as ethanol targets. Nonetheless, it is important to note that mechanistic concepts as outlined below are likely to apply as well to other membrane-associated signaling molecules.

Modulation of ion conductances and consequently of excitability is a key mechanism underlying ethanol effects on sympathetic neuronal activity [55], the function of the sympathoadrenal axis as well as on vascular reactivity [64]. Neuronal structures essential for blood pressure control such as the hypothalamic paraventricular nucleus and arterial baroreceptors express a divergent array of cation channels, which have been proposed as prominent targets for ethanol action [70,71]. These potential ethanol targets include also elements of mechanotransduction such as TRP (transient receptor potential) channel members of the TRPA, TRPV and TRPC subfamilies) or the epithelial Na^+^ channel (ENaC). Obviously, the spectrum of proposedly ethanol-sensitive ion channels in the brain and in peripheral neurons is highly diverse. Similar diversity is likely for ion channels, which govern vascular functions and reactivity. Most prominently, K^+^ channels expressed in smooth muscle and endothelial cells (BK and Kir channels) [65,69] but also Kv7 channels [23,55] and Ca^2+^ channels [72] have been suggested as a target of ethanol action in vascular tissues and most likely also in adrenal chromaffin cells [73]. Ethanol modulation of these channels appears as a plausible basis of ethanol toxicity as well as alcohol-induced hypertension. Among the currently considered molecular mechanisms by which ethanol is able to affect the function of ion channels, the primary one is the direct, reversible binding of ethanol or an ethanol metabolite to the target proteins. Indeed, there is reasonable consensus for a divergent set of ion channels to function as “ethanol receptors” being controlled by ethanol in a rather direct manner. Distinct binding pockets for the reversible coordination of ethanol have been identified and characterized not only for pentameric ligand-gated channels [57] but more recently also for K^+^ channels expressed in vascular cells [64,74,75] as well as in neurons associated with sympathetic tone [76,77]. Hence, hydrophobic alcohol coordination sites in different types of ion channels are increasingly recognized to mediate ethanol toxicity. A plausible alternative to ethanol action is the modulation of ion channel functions via altered physicochemical features of the lipid bilayer as a consequence of ethanol partitioning that needs consideration. Ethanol is known to interfere with the molecular organization of the lipid bilayer, specifically to disrupt certain cholesterol–lipid interactions and alter lateral pressure profiles in the membrane [52,53]. It appears reasonable to envision these effects of ethanol as a determinant of membrane-delimited signaling processes including ion channel functions. Nonetheless, there is, to date, little if any evidence for this concept with respect to the channels implicated in pathogenesis of hypertension. Another alternative concept of ethanol action is the molecular recognition of metabolites and mediators that are generated in response to ethanol metabolism by ion channels. This mechanism has indeed been proposed for ion channels relevant to the development of hypertension such as K^+^ channels and the epithelial Na^+^ channel (ENaC) [36,78], and the key role of ethanol metabolites was put forward with respect to observed disturbances in vascular reactivity as a consequence of alcohol consumption [69]. More recently, it has become evident that ethanol not only features the capacity to interfere with the lipid organization in biomembranes but is, in a similar manner, able to disturb protein–lipid switches that govern signaling processes in the membrane. Such lipid switches are for a large part based on reversible, electrostatic interactions between membrane lipids and signaling molecules, and are crucial for proper membrane recruitment as well as function of these signaling elements.

### 3.1. Lipid–Protein Switches in Signaling Molecules as a Target of Ethanol Action

**Figure 2:**
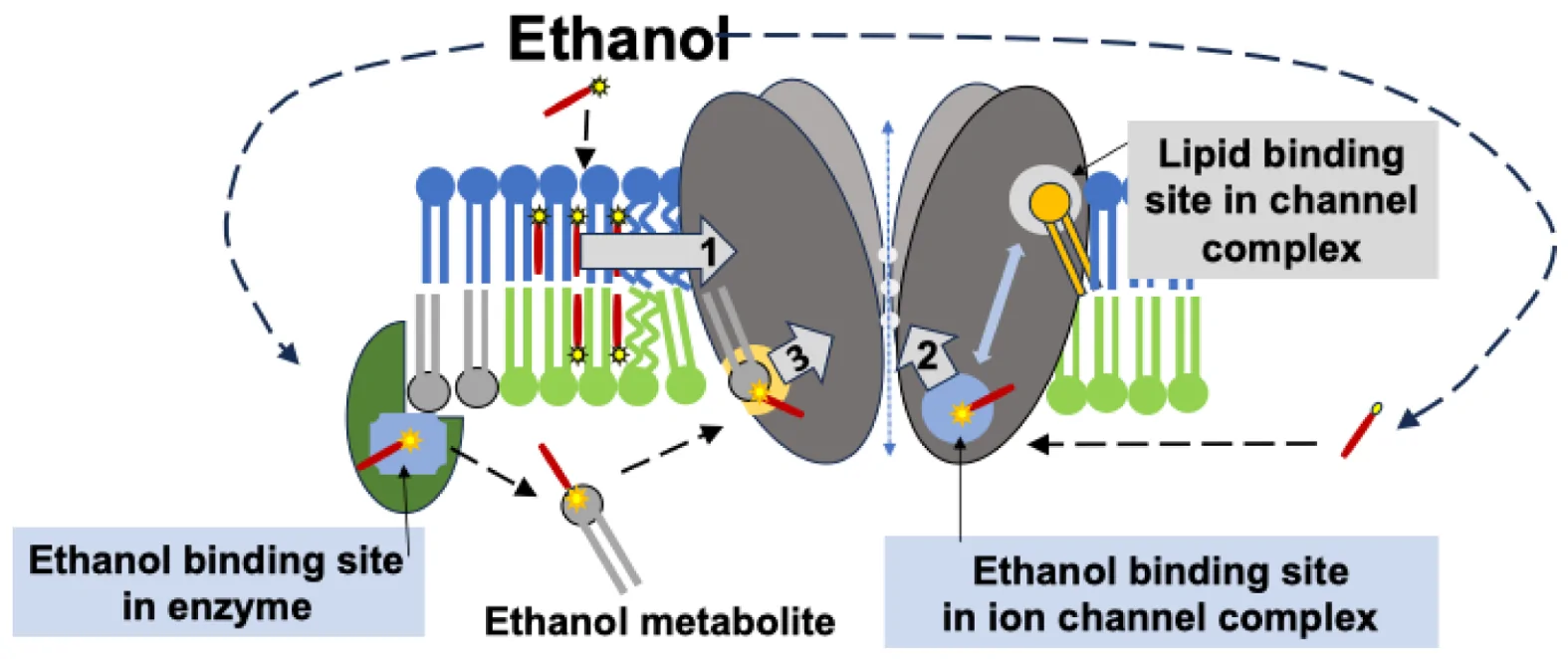
Cartoon illustrates an emerging concept of ethanol action on membrane signaling elements exemplified with ion channels as ethanol targets. Arrows indicate the hypothetical interaction of ethanol with divergent primary target structures associated with different modes of action (1,2,3). We hypothesize these mechanisms need consideration for a wide array of other membrane signaling systems. Besides integration of ethanol into the membrane bilayer structure and disturbance of bilayer-protein interactions (1; [53]), the existence of direct binding sites within ion channel complexes are established for a variety of channel families (2). Hydrophobic ethanol binding pockets in ion channels have been found to communicate with regulatory lipid coordination within channel complexes. Alternatively, ethanol may interfere with lipid regulation of channel functions after being metabolized at the plasma membrane to compete with regulatory membrane lipids (3). The binding of ethanol or its metabolites to hydrophobic sites may interfere with lipid regulation of ion channels either by allosteric coupling of hydrophobic regulatory sites or by direct disruption of lipid coordination (2). Documented examples for the allosteric coupling concept (2) are inwardly rectify K^+^ channels and large-conductance Ca^2+^-dependent K^+^ (BK) channels, while recently the two-pore K^+^ channel TREK-1 was reported as a target of the ethanol metabolite phosphatidylethanol, which proposedly competes with anionic lipid regulators (3; [63]). To date, compelling evidence for the interference of ethanol with structural and/or mechanical communication between channels and the lipid bilayer (“force from lipid” gating; illustrated in 1) is, to our knowledge, lacking although channels involved in mechanotransduction such as TRPA1 and TRPC6 have been reported as ethanol sensitive [84,87,88]. Of note, TRPV6 represents another lipid-regulated ion channel that is also a proposed ethanol target structure [89].

Most, if not all of the signaling processes, which have been proposed as targets of ethanol action, are “polymodal” and tightly controlled by protein–lipid switches as well established for K^+^ and TRP channels [79,80]. Reversible interactions between membrane lipids and ion channels are increasingly understood as key determinants of their signaling function. Lipid–protein interactions are not only essential to regulate membrane presentation and the molecular organization of ion channels within functional signaling units, but also to govern their gating and permeation. It is important to note that such regulatory protein–lipid interactions take place both at the peripheral interface between a channel complex and its annular lipid environment and within fenestrations and cavities of the protein complex representing “non-annular” regulatory sites [81]. Hence, the interference of ethanol with protein–lipid switches may affect ion channels and downstream signaling processes by different mechanisms. Ethanol may (i) disturb membrane targeting and assembly of channel complexes, (ii) compete with or disrupt the binding of lipids to the channels either directly or indirectly via metabolites, and/or (iii) affect essential regulatory protein–lipid interactions via allosteric mechanisms. Of these alternatives, the latter scenario is well documented for some hypertension-relevant ion channels including K^+^ channels as well as TRP channels [68]. Ethanol-sensitive ion channels demonstrated to harbor regulatory lipid coordination sites are Kv7 [55] and inwardly rectifying Kir3 channels [82,83], which are critically controlled by PIP_2_, while Kir3 as well as large-conductance Ca^2+^-activated K^+^ (BK) channels are both modulated by membrane cholesterol [74,75,83]. TRPC6, which has been suggested as an indirect target of ethanol action [84], is involved in renal as well as vascular pathologies and critically controlled by diacylglycerol (DAG), PIP_2_ and cholesterol. Interdependence/crosstalk between alcohol binding and lipid regulation of channels has been proposed with respect to anionic lipid regulators such as PIP_2_ and phosphatidic acid [63] as well as cholesterol [85]. For some hypertension-relevant ion channels, ethanol binding regions have been identified in proximity to regulatory lipid–protein interactions, and allosteric linkages between these critical sensor domains appear plausible. This concept is illustrated in the cartoon depicted in Figure 2, highlighting the functional coupling between an ethanol binding and a regulatory lipid coordination site. Of note, allosteric communication between regulatory sites, which coordinate lipids within ion channel complexes has recently been observed for TRPC channels [86]. Intriguingly, not only ethanol but also lipid–ethanol metabolites such as the PLD2 product phosphatidylethanol proposedly compete with lipids at certain regulatory sites in ion channels. A modulatory role of phosphatidylethanol has been suggested for two-pore K^+^ channels (TREK-1) in the fruit fly [63], schematically depicted in the cartoon shown in Figure 2. The alcohol metabolite may interfere with the binding of anionic lipid regulators such as phosphatidic acid at their coordination sites in the channel complex. Notably, the involvement of two molecular recognition sites, one localizing in the metabolizing enzyme and a second site residing in the target channel, enable the discrimination between alcohols featuring aliphatic chains of different length. These data prompt further investigations to test for the potential competition of ethanol or ethanol metabolites with regulatory lipid coordination in other toxicity- and disease-relevant ion channels and enzymes. Better understanding of the molecular mechanism(s) by which ethanol affects protein–lipid switches and elucidation of the role of the membrane lipid composition as a potential determinant of ethanol pathology may pave the way towards new strategies for the prevention and therapy of alcohol consumption-associated diseases.

### 3.2. Lipid–Protein Switches as an Emerging Target Involved in Ethanol-Induced Disturbances in Ca^2+^ Homeostasis

Acute and chronic toxicity as well as pathogenic features of ethanol reportedly involve the disruption of cellular Ca^2+^ signaling processes, as documented for astroglia, vascular cells and adrenal chromaffine cells [72,73]. Ethanol-induced impairment of Ca^2+^ homeostasis, specifically the distortion of Ca^2+^ signaling and exocytosis in adrenal chromaffin cells are likely relevant for the pathogenesis of hypertension. Consistent with the current mechanistic concepts for ethanol actions (as outlined above), disruption of Ca^2+^ signaling is hypothesized as based on either the direct binding of ethanol to hydrophobic sites in Ca^2+^ transport systems or critical regulatory proteins, or on the modulation of Ca^2+^ transport processes by oxidative stress generated in response to ethanol metabolism. Nonetheless, productive Ca^2+^ signaling in cells indispensably requires spatiotemporally precise, coordinated interactions between proteins and membrane lipids to enable proper signaling functions [90,91]. This applies in particular to STIM1-Orai-mediated (store-operated) Ca^2+^ signaling, which has been suggested as a hypertension-relevant target of ethanol action in vascular cells [75]. STIM-Orai-mediated Ca^2+^ signaling has been recognized as strictly dependent on membrane lipid composition and lipid–protein interactions [92]. As in many other Ca^2+^ signaling pathways, electrostatic protein–lipid interactions are a key switch in cellular control of this signaling process. The reported propensity of ethanol to interfere with lipid switches, including molecular mechanisms involved in reversible recruitment of signaling proteins into specific membrane domains, is exemplified by the reportedly direct impact of ethanol on targeting and activation of the presynaptic zone protein MUNC-13 [93]. Collectively, the current understanding of the critical role of spatiotemporally precise positioning of Ca^2+^ signaling elements within membrane nanodomains and the increasingly recognized potential of ethanol to interfere with protein–lipid interactions involved in membrane assembly of signaling complexes, prompt for further evaluation of the pathological significance of ethanol-triggered disruption of lipid switches involved in cellular Ca^2+^ homeostasis. As for K^+^ channels, the role of ethanol-sensitive Ca^2+^ handling proteins needs consideration for both ethanol actions on central blood pressure control mechanisms and local vasoreactivity. With respect to the latter aspect, it is important to appreciate that ethanol’s effects on vascular reactivity feature striking concentration dependence with divergent outcomes for vascular tone dependent on the dose and duration of ethanol administration [94]. The complex interference of ethanol with vasoregulation is likely to involve its impact on Ca^2+^ signaling processes within the vascular bed [94,95]. It will be a particular challenge to decipher the role of ethanol interference with lipid–protein switches in the context of this complex Ca^2+^-dependent modulation of vasoreactivity.

## 4. Alcohol and Membrane Lipids—A Partnership in Crime to Promote Arterial Hypertension

Alcohol-induced hypertension is based on a complex scenario of organ malfunctions, proposedly originating from altered cellular communication within divergent tissues and organs. A large number of pathways have been identified, each potentially serving as a critical mechanistic link between the molecular action of ethanol on its cellular targets and the impairment of cell signaling and alcohol-induuced hypertension. To date, neither the significance and quantitative contribution of such candidate pathways for hypertension development nor their interdependency or pathological synergy is understood. The design of clinically useful, targeted interventions will therefore require either a profound quantitative understanding of the complex network of ethanol-induced cellular malfunctions leading to hypertension or, alternatively, the identification of a mechanism of ethanol action, which is common for most or all relevant target cell types. Here we summarize evidence for the emerging concept of a potentially druggable mechanism, based on a generic interference of ethanol with regulatory lipid–protein interactions in signaling molecules. The key role of membrane lipids and lipid mediators in ethanol actions has long been recognized, specifically the role of the arachidonic acid metabolite TXA_2_ in ethanol-induced vascular hypercontractility [50]. This mechanistic aspect is also highlighted in Figure 1. Nonetheless, not only the ethanol-induced modulation of lipid, specifically arachidonate metabolism, needs consideration. Over the past decades, the pivotal role of communication between membrane lipids and membrane-resident signaling elements including receptors, transporters, ion channels, scaffold as well as adaptor molecules has been convincingly demonstrated. Structural biology and computational modeling approaches provided evidence for a regulatory role of dynamic lipid coordination within signaling complexes, which operate at or in cell membranes. In this context, protein–lipid interactions govern membrane-delimited signaling processes by reversible binding of lipid ligand(s) to signaling molecules or by an impact of lipids on membrane recruitment and retrieval of signaling elements [96]. Recent evidence suggests these mechanisms are of relevance for biological actions of ethanol. Hence, the mechanistic picture of ethanol toxicity and pathogenicity in humans has gained another level of complexity. Besides ethanol binding to distinct hydrophobic sites at molecular targets and/or modulation or disruption of signaling-relevant features of the lipid bilayer, ethanol may critically interfere with the regulation of signaling complexes by their lipid environment, recently termed the “functional paralipidome” [97]. A paradigm for this emerging concept of ethanol action is represented by ion channels, which have been characterized as both ethanol sensitive and regulated by reversible interactions with membrane lipid. Probably the most convincing example of signaling elements featuring lipid-dependent ethanol modulation are inwardly rectifier K^+^ channels [85] and voltage- and Ca^2+^-dependent K^+^ channels (BK) [98]. Accumulating evidence suggests that the lipid regulation of diverse signaling pathways may constitute a prominent target of ethanol with respect to the etiology of hypertension. Disruption of lipid–protein switches by ethanol or ethanol metabolites may compromise the functions of signaling elements, impair their lipid-dependent membrane presentation and (or) spatiotemporal organization thereby leading to aberrant neuronal functions and vascular hyperreactivity as well as remodeling of the vascular bed and ultimately arterial hypertension. Of note, the discussed concept of ethanol interference with lipid switches in signaling processes deserve consideration for other alcohol use disorders as well.

## 5. Conclusions

- Alcohol-indued hypertension is based on a complex network of dysregulations impairing both central blood pressure control and peripheral vascular functions. The primary molecular targets mediating disease etiology are likely multifunctional, ubiquitously expressed signaling molecules.
- Emerging evidence suggests ethanol as a potent modulator of lipid–protein switches, which govern critical membrane signaling elements as exemplified for ion channels. This led us to propose cellular lipid–protein switches as a prevalent and potentially druggable target involved in the etiology of alcohol-induced hypertension.
- A large array of ion channels that are expressed in both the nervous system and the vascular bed such as K^+^ and TRP channels, appear to be affected by ethanol or its metabolites in a strikingly lipid-dependent manner. This represents a novel aspect of ethanol toxicology and needs consideration as a basis for the development of efficient, targeted therapies or strategies for the prevention of alcohol consumption-associated pathologies.
- Further, detailed analysis of the dependency of ethanol actions on membrane lipid composition and lipid metabolism may help to advance our understanding of the complex ethanol dose-dependency, gender and ethnic diversity seen in epidemiology of drinking-associated diseases including alcoholic hypertension.

Limitations: There are certain limitations of this narrative review on the mechanism of alcohol-induced hypertension focusing on membrane lipids and ion channels of vascular smooth muscle. Formal PRISMA/AMSTAR-2 evaluation is lacking for the meta-analyses cited in the introduction of this review, which are limited to articles written in English, and thus there is a risk of language bias.

## Figures and Tables

**Table 1:** Relationship between alcohol consumption and hypertension in meta-analysis of previous cohort studies.

Reference Number	Number of Analyzed Articles	Number of Analyzed Participants	Countries (Number of Analyzed Articles)	Follow-Up Period (Years)	Results (Relationship Between Alcohol Consumption ** and Incidence of Hypertension; Ethnic Difference ***)
[5]	12	27,603 (patients with HT)	US (7), Japan (4), Korea (1)	4~24	Men: positive (dose-dependent) Women: inverse (<5 g/day), positive (>5 g/day); more prominent elevation by heavy drinking compared with men Asian men > non-Asian men
[6]	16	227,656 (overall); 56,348 (patients with HT)	US (10), Japan (5)	4~20	Men: positive (dose-dependent) Women: inverse (<20 g/day), positive (>21 g/day)
[7]	20	361,254 (overall) 90,160 (patients with HT)	US (9), Europe (2), Japan (4), other Asia (4), Turkey (1)	3.9~20	Men: positive (dose-dependent) Women: positive (>24 g/day); not different (12~24 g/day)
[8]	11 *	86,188 (overall)	US (3),Germany (1), Japan (5), China (1), Korea (1)	0.9~22	Men: positive (dose-dependent) Women: positive (significant only in heavy drinkers of >60 g/day) Asian men > Western men
[9]	23	>600,000 (overall) >45,000 (patients with HT)	US (8), Europe (3), Japan (4), China (5)	2~22	Men: positive (dose-dependent) Women: positive (only in drinkers with alcohol of >12 g/day); more prominent elevation by heavy drinking compared with men Western people = Asian people; White people > black people
[10]	22 (31 studies)	414,477(overall) 89,734 (patients with HT)	US (17), Europe (4), Australia (1), Japan (3), other Asia (5), Mauritius (1)	9.01 (average)	Men: positive (dose-independent) (more prominent elevation in light drinkers (10–30 g/day) than in heavy drinkers (30–50 g/day)) Women: J-shaped (no significant difference in light drinkers (10 g/day) compared with nondrinkers) Black people > Asians and white people

HT, hypertension. *, including one case–control study. **, alcohol consumption per day (g/day). ***, >, higher sensitivity to alcohol; = similar sensitivity to alcohol. Ten grams of alcohol is found in 250 mL of beer that contains alcohol at 5%.

## Data Availability

No new data were created or analyzed in this study. Data sharing is not applicable to this article.
